# Milk phospholipid-coated lipid droplets modulate the infant gut microbiota and metabolome influencing weight gain

**DOI:** 10.1186/s40168-025-02106-w

**Published:** 2025-05-14

**Authors:** Simone Zuffa, Christophe Lay, Elizabeth A. Wimborne, Arabella Hornung Rodriguez, Yi Wu, Franklin L. Nobrega, Nana Bartke, Anita C. S. Hokken-Koelega, Jan Knol, Guus Roeselers, Jonathan R. Swann

**Affiliations:** 1 Skaggs School of Pharmacy and Pharmaceutical Sciences, University of California San Diego, San Diego, USA; 2 Department of Metabolism, Digestion and Reproduction, Faculty of Medicine, Imperial College London, London, UK; 3 Danone Research & Innovation, Precision Nutrition, D-Lab, Singapore, Singapore; 4 Faculty of Medicine, School of Human Development and Health, University of Southampton, Southampton, UK; 5 Faculty of Life Sciences, School of Biological Sciences, University of Southampton, Southampton, UK; 6 Danone Research & Innovation, Utrecht, The Netherlands; 7 Erasmus University Medical Centre, Sophia Children’s Hospital, Rotterdam, The Netherlands

**Keywords:** Formula milk, Lipids, Milk fat globules, Metabolomics, Microbiome, Microbiota, Breast milk, Triglycerides, Infant, BMI

## Abstract

**Background:**

The supramolecular structure and composition of milk fat globules in breast milk is complex. Lipid droplets in formula milk are typically smaller compared to human milk and differ in their lipid and protein composition. These droplets play an important role in gut and immune maturation, and their components possess antimicrobial and antiviral properties. Here, the influence of a concept infant formula (IF) containing large milk phospholipid-coated lipid droplets on the maturation of the infant microbiota, metabolome, and weight gain in the first year of life was investigated.

**Results:**

Formula-fed infants were randomized to receive either a standard IF (Control) or a Test formula containing large milk phospholipid-coated lipid droplets (Test) until 17 weeks of age. A breast-fed Reference group was also investigated. At 3 months of age, several taxa identified as opportunistic pathogens (e.g., *Enterobacter*, *Klebsiella*, *Enterococcus*, *Streptococcus*) were less abundant in the Test stools compared to Control, while an enrichment of the butyrate-producing Ruminococcaceae and Lachnospiraceae was observed. These findings indicate that the Test formula resulted in gut microbiota maturation trajectories more comparable to healthy breast-fed infants. This was accompanied by variation in several fecal and plasma metabolites at 3 months of age related to gut microbial metabolism including bile acids, hippurate, phenylacetylglycine, trimethylamine, and various lipids and fatty acids. At 12 months, measures of subcutaneous fat and body mass index (BMI) were significantly higher in infants receiving standard IF compared to those receiving breast milk. However, this weight gain and adiposity was attenuated in the Test group infants.

**Conclusions:**

The presence of large phospholipid-coated lipid droplets in formula milk positively influenced the development of the infants’ gut microbiota, their metabolomic profiles, and their body composition to more closely resemble breast-fed infants compared to standard IF. These droplets may further enhance the restriction of pathogenic bacteria seen with standard infant formula and suggest a potential impact on infant metabolic programming that may contribute to physiological development.

Video Abstract

**Supplementary Information:**

The online version contains supplementary material available at 10.1186/s40168-025-02106-w.

## Background

Human milk is a highly complex biofluid containing hundreds of different biologically active components, such as carbohydrates, proteins, and lipids, which support the growth and development of infants. Lipids are an important source of energy for the developing infant, accounting for approximately 55% of the total caloric intake of newborns [1], and are produced in the mammary glands and packed to form milk fat globules (MFGs) [2]. These globules have an internal core rich in triglycerides and an external membrane, also known as milk fat globule membranes (MFGM), rich in phospholipids, sphingomyelin, glycolipids, cholesterol, and (glyco)proteins [3]. MFGs play an important role in development, promoting gut maturation by inducing epithelial cell proliferation and differentiation, increasing tight junction protein expression, and are involved in the development of the immune system [2]. MFGM components are considered to possess antimicrobial and antiviral properties [4, 5], and intervention studies in pediatric populations have reported the positive impact of MFGM supplementation on immunity (i.e., reduced incidences of infections) and cognitive outcomes [6, 4, 5, 7]. Although interactions between dietary lipids and the gut microbiota have been proven to influence host physiology, little is known about how MFGs influence the development of the intestinal microbiota and their metabolic interactions with the human host and their implications for the phenotypic maturation of the holobiont.

Currently, commercialized infant formula milk derived from cow, goat, or plant-based milk typically contains smaller lipid droplets (mode diameter 0.4 μm) compared to the human MFGs (mode diameter 4.2 μm), which are primarily coated by proteins at their surface [1]. Size, structure, and composition of MFGs are believed to be important, specifically to increase contact surface and improve absorption [8]. Recently, a novel infant formula with large milk phospholipid-coated lipid droplets (Nuturis®) was developed to resemble human MFGs in size and partially in structure and composition. These dairy and vegetable oil lipid droplets have a mode diameter of 3–5 μm and consist of a triglyceride core enclosed in a layer of phospholipids, sphingomyelin, glycolipids, cholesterol, glycosylated proteins, and milk proteins. The safety and tolerance of an infant formula containing these large lipid droplets have been demonstrated in healthy term born infants [1, 9]. Preclinical studies have also demonstrated that early-life consumption of these lipid droplets prevent fat accumulation when mice were subsequently challenged with a Western-style diet during adulthood [10]. This is noteworthy as formula-fed infants have increased odds of developing obesity in later life compared to breast-fed infants with well-established differences in the colonization patterns of their gut microbiota [11, 12].

As an exploratory objective of a randomized, double-blind controlled multicenter clinical trial, the current study investigates whether a concept infant formula containing large, milk phospholipid-coated lipid droplets with a mixture of dairy and vegetable lipids alters the maturation of the intestinal microbiota and impacts infant weight gain and body composition in the first year of life compared to standard infant formula [9]. An integrated microbial (16S rRNA gene sequencing) and metabolomic (^1^H nuclear magnetic resonance (NMR) spectroscopy and liquid chromatography-mass spectrometry (LC–MS)) profiling approach was applied to longitudinally collected stool and plasma samples over the first year of life to characterize the evolution of microbial-host interactions and how these are shaped by early-life nutrition.

## Methods

### Study population and sample collection

This exploratory, randomized, double-bind controlled study was conducted in Belgium, The Netherlands, France, and Singapore between October 2012 and December 2014 [9]. This trial was registered in the Dutch Trial Register (www.trialregister.nl) as NTR3683. Ethical approval was obtained for the study per country whereupon also approval of the independent local ethics review boards of all the participating centers. Informed consent was provided by the legal guardians. A total of 311 infants were enrolled in the study. Of these, 223 were formula-fed, and 88 were breast-fed at recruitment. Breast-fed infants represented the Reference group, and their parents were instructed to exclusively breastfeed them until at least the 13 th week of age and preferably for the whole duration of the study. Formula-fed infants were randomized into two different groups: Test and Control. The Control group (*n* = 108) received a cow milk-based formula supplemented with prebiotics (0.8 g/100 mL of scGOS/lcFOS). The Test group (*n* = 115) received the same prebiotic containing (0.8 g/100 mL of scGOS/lcFOS) cow milk-based formula supplemented with large, milk phospholipid-coated dairy-vegetable lipid droplets (Nuturis®). This Test product (Nuturis®) contained a mixture of vegetable and dairy lipids (52:48 ratio) and for this reason also higher levels of sn-2 palmitic acid. The control product was exclusively vegetable-oil based. Both products contained additional fish and algal oils as a source of long-chain polyunsaturated fatty acids. Due to the application of an adapted manufacturing process, the lipid droplets in the Test product had a volume-based mode diameter of 3–5 M, while the lipid droplet size of the Control product had a volume-based mode diameter of approximately 0.5 M. The intervention lasted from enrollment (infants age ≤ 35 days) until the infant age of 17 weeks, with infants from each distinct group following any feeding approach after that period. Anthropometric data were recorded at birth and each visit, enrollment (Visit 1), at 3 months of age (Visit 2), and at 1 year of age (Visit 3) and included weight, length, and head circumference among others. These data were used to calculate the body mass index (BMI) and weight for length (WFL) of the infants. Additionally, measurements of subcutaneous fat were recorded, such as triceps skin-fold thickness, subscapular skin-fold thickness, biceps skin-fold thickness, and suprailiac skin-fold thickness. Stool samples were collected each visit, while plasma samples were collected exclusively at Visit 2 (3 months of age).

From the 311 recruited infants, gut microbiota profiling and fecal and plasma metabolomic analysis were performed on a subset of 164 participants whose stool and plasma samples were available for profiling from the clinical study’s biobank. Demographics of the analyzed subjects showed no differences in sex composition between the three different groups (Supplementary Table 1). All groups had comparable anthropometrics and gestational ages (ANOVA, *p* > 0.05; Supplementary Fig. 1).

### Fecal microbiota analysis

DNA was extracted from the stool samples as previously described [13]. Primers Bact- 0341 F and Bact- 0785R were used to amplify the region V3–V4 of the 16S rRNA gene [14]. Amplicons were sequenced on an Illumina MiSeq instrument (Illumina, San Diego, CA, USA) as previously described [15]. QIIME 1.9.0 was used to generate the Operational Taxonomic Units (OTUs) table from the sequencing data [16]. Quality control filters were applied as previously described [17]. De novo OTU picking was performed with the USEARCH algorithm using a 97% sequence identity [18]. The SILVA database (release version 1.1.9) was used for taxonomy assignment [19]. Singletons and low-abundant OTUs with a relative abundance < 0.002% were excluded for downstream analysis.

Rarefaction curves reached a plateau, indicating good coverage of the gut microbial communities, and sequencing depths were comparable between groups, approaching negative binomial distributions. Data were investigated via the phyloseq framework and collapsed at genus level before investigation using a compositional data analysis (CoDA) workflow. Zeroes in the OTU count table were imputed with Bayesian-multiplicative replacement and then center log ratio (CLR) transformed using the “CoDaSeq” package. Unsupervised and supervised dimensionality reduction analyses (principal components analysis (PCA) and partial least squares-discriminant analysis (PLS-DA)) were performed with the “mixOmics” package. Group differences were calculated with PERMANOVA on the Aitchison distances using the adonis function from the “vegan” package. Alpha diversity was calculated at each timepoint for each group and compared using Kruskal–Wallis test followed by Wilcoxon test and Benjamini-Hochberg (BH) correction. Three different alpha-diversity metrics were investigated: observed OTUs, Chao1, and Shannon. PLS-DA model performances were evaluated through the classification error rate (CER) metric using leave-one-out (LOO) cross validation provided by the perf function of “mixOmics.” Features with variable importance projection (VIP) scores > 1 were considered significant for group separation. Univariate visualization of taxa of interest was generated on the CLR-transformed counts. Top taxa differentiating groups were also plotted as log ratios. ANCOM-BC2 differential abundance analysis was performed adjusting for mode of delivery and antibiotic exposure.

#### ^1^H NMR spectroscopy

Fecal aliquots (50 mg) were defrosted on ice. Approximately, 10 zirconia beads of 1-mm diameter (BioSpec Products, US) and 700 µL of demineralized water were added to each homogenization tube. Samples were homogenized using a Precellys 24 homogenizer (Bertin Instruments, FR) with 2 × 6500 rpm in a 2-min program (2 × 40 s homogenization, with a 20-s interval). After homogenization, samples were centrifuged at 10,000 g for 20 min at 4 °C. In new 1.5-mL Eppendorf, 630 µL of fecal water (supernatant) was collected and supplemented with 70 µL of phosphate buffer (1.5-M KH_2_PO_4_, 2-mM NaN_3_, 1% 3-(trimethylsilyl)− 2,2,3,3-tetradeuteropropionic acid (TSP) solution, pH 7.4). Samples were vortexed and centrifuged at 10,000 g for 5 min at 4 °C. Finally, 600 µL of the samples was transferred into 5-mm NMR tubes for ^1^H NMR spectroscopic analysis. Samples were loaded into a refrigerated SampleJet robot (Bruker), and standard NOESY and JRES experiments were run in automation at 300 K on a Bruker 600-MHz UltraShield spectrometer (Bruker Biospin, Karlsruhe, Germany). A standard one-dimensional pulse sequence with saturation of the water resonance was applied (RD 90°-t1 90°-tm 90°-acquire FID, with RD set at 2 s and tm at 100 ms). For each spectrum, 8 dummy scans, followed by 64 scans with 32 K data points and a spectral width of 20,000 Hz, were acquired.

Spectra were automatically corrected for phase and baseline distortion and then calibrated to the TSP singlet (0.00) in TOPSPIN 3.2 (Bruker, Germany). The obtained spectra were digitized in MATLAB 2019b using IMPaCTS [20]. TSP (− 0.2 to 0.2 ppm) and water (4.7 to 4.9 ppm) regions were removed from the spectra. Peaks were manually aligned using recursive sample-wise peak alignment (RSPA) and then normalized using probabilistic quotient normalization to account for possible dilution factors. PCA was used for preliminary unsupervised analysis to identify possible outliers. Pairwise supervised analysis between groups was performed on full resolution spectra using PLS-DA. All peaks from the full resolution spectral data were annotated using an in-house database and HMDB (http://www.hmdb.ca/). STOCSY was used to identify peaks belonging to same metabolite (Pearson correlation > 0.8). Single representative peaks from the annotated metabolites were integrated and used for further analysis.

#### Targeted UPLC-MS analysis

Plasma, fecal, and infant formula samples were analyzed using the commercial Biocrates MxP® Quant 500 kit (Biocrates Life Sciences AG, Innsbruck, Austria) with a Xevo G2-XS QToF (Waters, Germany) mass spectrometer. Briefly, 10 µL of samples was added to the 96-well Biocrates plates, where the derivatization and extraction steps were performed. Two separate analytical runs followed, combining both liquid chromatography (LC) and flow injection analysis (FIA) to tandem mass spectrometry. FIA-MS was used to analyze the lipid classes, and a semiquantitative output was provided. Obtained data from the analytical runs were imported in the companion MetIDQ™ software which performed automatically peak detection, alignment, identification, and normalization. Tables with quantified metabolites were used for statistical analysis. Limits of detection (LOD) were calculated by the software, and metabolites that were below the LOD in > 80% of the samples were excluded from downstream analysis. For those metabolites with LOD values < 80% of samples, the < LOD values were replaced with the LOD for that compound divided by the square root of 2. Metabolism indicators were also calculated from the plasma and stool metabolic profiles.

Integrated peaks from the NMR spectra were log transformed before analysis, while obtained quantified values from the MetIDQ™ software were not transformed or adjusted, since the software automatically applies batch correction to the data. Ordination and relevant features separating the groups were identified using PCA and PLS-DA models as previously described for the microbiome analysis using the “mixOmics” package. Pairwise analysis of the metabolism indicators was performed with volcano plots. Outliers were identified using the Mahalanobis distance for multivariate data analysis and removed; the threshold was set to 5 × 10 [13]. Wilcoxon rank-sum test was used to calculate the *p*-values, and these were then adjusted using FDR correction (*q* = 0.05). A fold change threshold of 1.5 and − 1.5 was used.

#### Data integration

Spearman and Pearson correlation coefficients were calculated between bacteria, fecal and plasma metabolites, and anthropometrics to identify relations between biological measurements and growth using the “Hmisc” package. Correlations with *p* < 0.05 were considered as significant, and adjusted *p*-values were also calculated with BH correction. The different omics blocks from the samples collected at Visit 2 (3 months of age) were integrated using the DIABLO framework from the “mixOmics” package [21]. Only participants for which fecal, plasma, and microbiota profiles were available were retained in the analysis. A design matrix with 0.1 correlation coefficient between the block was chosen to maximize the discriminatory power. Model performance (CER) was assessed using LOO cross-validation. After dimensionality reduction, projections were visualized with scatter plots (ellipses explaining 95% confidence interval), and a circos plot was generated to visualize the correlations between the variables of the different omics block. The correlation cutoff was set at 0.6, and variables influencing both the first and the second components were included.

#### Statistical analysis

All statistical analysis was performed on R version 4.0.2 (R Foundation for Statistical Computing, Vienna, Austria). Available metadata were explored using analysis of variance (ANOVA) or Kruskal–Wallis test when comparing multiple groups, depending on data distribution. Pairwise comparisons were performed with Wilcoxon test and BH correction or Tukey’s HSD test. Significance was considered for *p*-value < 0.05 or *p*-adjusted < 0.05 if not otherwise stated. Categorical data were analyzed with the Fisher’s exact test. One outlier identified using Grubbs’ test, from the Test group, was removed for the SUMSK analysis.

## Results

**Fig. 1:**
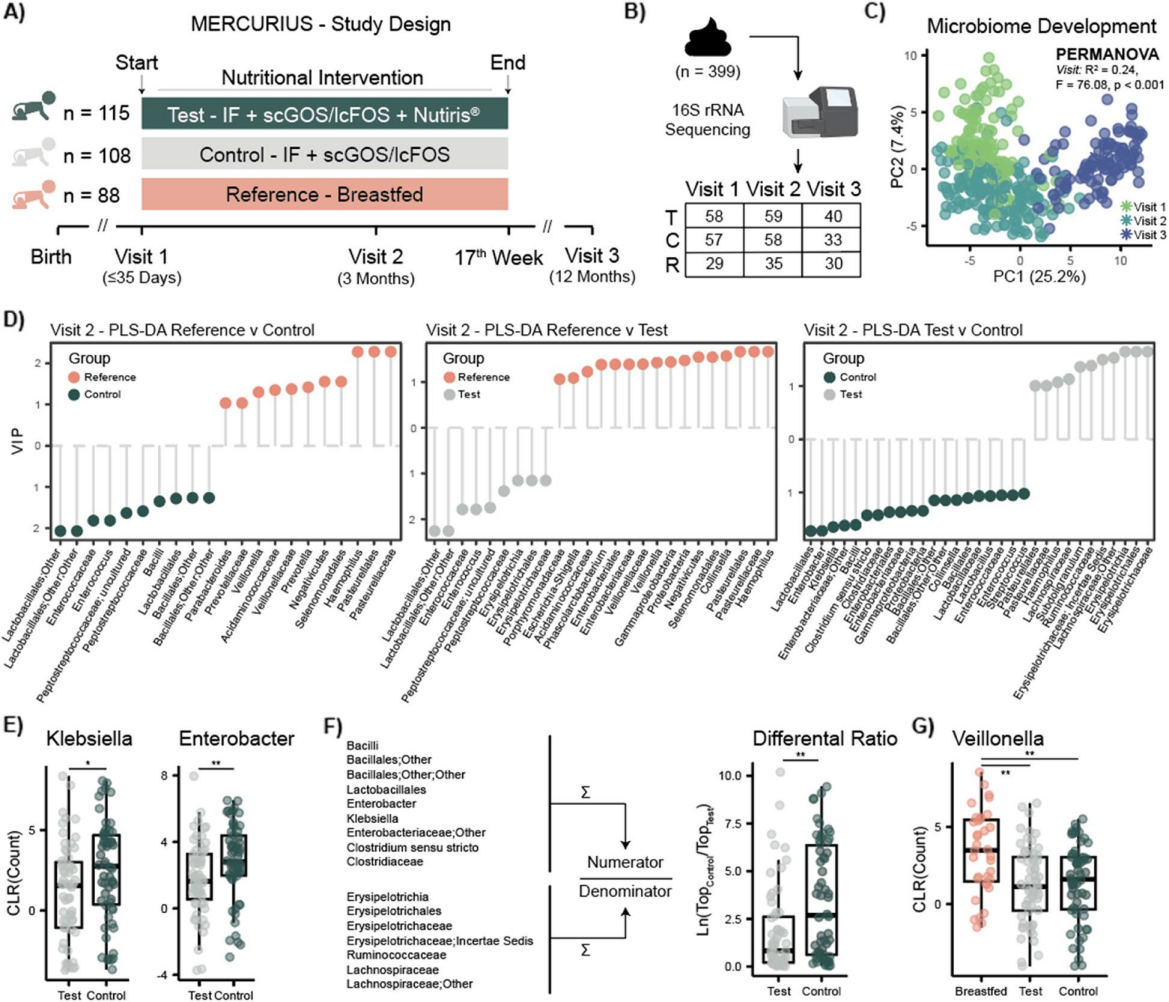
Fecal microbiota profile comparisons at 3 months of age. **A** Formula fed infants were randomized into two groups: Test (*n *= 115), which received IF supplemented with 0.8 g/100 mL of scGOS/lcFOS and Nuturis^®^, and Control (*n* = 108), which received IF supplemented with only scGOS/lcFOS. A Reference group of breastfed infants was also enrolled. The nutritional intervention lasted up to the 17^th^ week of life, with infants following any feeding approach subsequently. Fecal samples were collected at Visit 1, 2, and 3. **B** Number of fecal samples (*n* = 399) available for 16S rRNA sequencing from the Test (T), Control (C), and Reference (R) groups. **C** PCA of CLR transformed counts of all fecal samples acquired via 16S rRNA sequencing. A strong cluster based on visit, and thus age of infants, was observed (PERMANOVA, R^2^ = 0.24, F = 76.08, *p* < 0.001). **D** Pairwise PLS-DA models of the CLR transformed bacterial counts were constructed to investigate taxa driving group differences at Visit 2. Reference infants presented a higher abundance of Pasteurellales, Pasteurellaceae, Veillonellaceae, *Veillonella*, and *Haemophilus* compared to both Test and Control infants. Test group was associated with a reduced abundance of *Enterococcus*, *Enterobacter*, and *Klebsiella* compared to Control infants. All PLS-DA models had CER < 0.5 and features with VIP > 1 were retained for visualization. **E** Univariate visualization of *Klebsiella* and *Enterobacter* differential abundances between Test and Control. Significance tested via Wilcoxon test. **F** Natural log ratios generated from features either associated with Test or Control. Significance tested via Wilcoxon test. A linear model was built to investigate the possible effect of covariates: lm(Ratio ~ Group + Sex + Delivery mode + Antibiotics + Weight). **G** Breastfed infants presented higher levels of *Veillonella*. Pairwise Wilcoxon followed by BH correction. Asterisks in score plots represent group centroids. Boxplots show first (lower) quartile, median, and third (upper) quartile. Significance: * *p* < 0.05, ** *p* < 0.01

A total of 313 infants were screened for the MERCURIUS clinical trial (NTR3683). Of these, two were ineligible resulting in a final study population of 223 formula-fed and 88 breast-fed infants (Fig. 1A). The breast-fed infants (Reference) were on average fully breast-fed until the 13 th week of life and preferably until 17 weeks of age. Mean duration of breastfeeding was 128 days (95% *CI*: 120–135 days). Formula-fed infants were randomized into two different groups; the Control group (*n* = 108) received a cow milk-based infant formula supplemented with prebiotics (0.8 g/100 mL of scGOS/lcFOS), while the Test group (*n* = 115) received a nutritionally similar infant formula supplemented with the same prebiotics (0.8 g/100 mL of scGOS/lcFOS) and large, milk phospholipid-coated lipid droplets. The Test product contained a mixture of vegetable and dairy lipids (52:48 ratio), while the Control product was vegetable-oil based. Both contained additional fish and algal oils. Due to the application of an adapted manufacturing process, the lipid droplets in the Test product had a volume-based mode diameter of 3–5 M, while the lipid droplet size of the Control product had a volume-based mode diameter of approximately 0.5 M. The nutritional intervention lasted from enrollment (infants age ≤ 35 days) until the 17 th week of life (~ 4 months), with all infants following any feeding approach after that period. Anthropometric data were recorded at birth, enrollment (Visit 1; mean age 12.78 days ± 10.6), 3 months (Visit 2; mean age 90.31 days ± 3.34), and at 12 months of life (Visit 3; mean age 369.08 days ± 9.69). Stool and plasma samples available from a subset of infants (*n* = 164) were analyzed to investigate their microbial and metabolomic profiles. Study infants were predominantly vaginally born, ranging from 68% in the Test group to 77% in Control group, and Caucasian (no significant differences between the three groups; demographic data provided in Supplementary Table 1). Additionally, no significant differences in antibiotic exposure during the first year of life were observed between the three groups (Fisher’s exact test, *p* > 0.05), ranging from 15 to 26%.

### Test formula positively influences fecal microbiota composition

Microbial profiling was performed on stool samples (*n* = 399) collected from a subset of 164 infants at enrollment (Visit 1), 3 months (Visit 2), and 1 year of life (Visit 3), using *16S rRNA* gene sequencing (Fig. 1B). An average depth of 317,229 reads per sample was obtained, and a total of 289 unique OTUs were identified. Principal components analysis (PCA) of the center log ratio (CLR) transformed OTUs collapsed at genus level was used for ordination. Clear age-dependent variation in the fecal microbial profiles was observed (PERMANOVA, *R*^2^ = 0.24; *F* = 76.98, *p* < 0.001; Fig. 1C). OTUs correlating with the first principal component (absolute correlation > 0.6) showed that samples from 1-year-old infants had a higher abundance of butyrate-producing Bacillota, previously Firmicutes, such as Ruminococcaceae and Lachnospiraceae and lower amounts of Pseudomonadota, previously Proteobacteria, compared to samples from the first few weeks of life (Supplementary Fig. 2 A). Alpha diversity was calculated for each group at each timepoint. At enrollment, higher alpha diversity was noted in the Reference group compared to the other groups for both observed OTUs and Chao1, but not for Shannon metrics (Supplementary Fig. 2B). However, this was explained by the significantly higher enrollment age of the Reference infants compared to the other two study groups (mean age in days (± SD): Reference, 18.3 ± 11.12; Test, 11.5 ± 9.85; Control, 9.86 ± 9.40). At 3 months, formula-fed infants from the Test group displayed a significantly higher alpha diversity for the observed OTUs metric compared to the Reference group (*p* = 0.0016) and a trend to be higher compared to the Control group (*p* = 0.072). Nevertheless, no differences were observed for the Chao1 and Shannon index. PCA of CLR-transformed OTUs counts was used to investigate gut microbial profiles at each timepoint (Supplementary Fig. 2 C). The intervention groups consistently separated from each other at all timepoints (PERMANOVA, *p* < 0.05) with maximum observed variance occurring at 3 months of life, during the nutritional intervention.

Pairwise partial least squares-discriminant analysis (PLS-DA) models were constructed on the stool microbial profiles at each timepoint to investigate microbial variation between the groups. Consistent with conventional use, OTUs with a variable importance in projection (VIP) > 1 were considered significantly discriminating the groups [22]. At enrollment, infants in the Test group had a higher abundance of Erysipelotrichia, Erysipelotrichales, Erysipelotrichaceae, Actinomycetota, Bifidobacteriales, Bifidobacteriaceae, and *Bifidobacterium*, while those in the Control group presented a greater abundance of Firmicutes, Bacilli, Lactobacillales, Streptococcaceae, *Streptococcus*, Planococcaceae, *Planomicrobium*, and *Haemophilus* (classification error rate [CER] = 0.48, full list of VIPs in Supplementary Table 2). At 3 months of age, stool samples from infants in the Test group presented greater abundance of Erysipelotrichia, Erysipelotrichales, Erysipelotrichaceae, Lachnospiraceae, and Ruminococcaceae compared to Control (Fig. 1D). Conversely, Control stools had more Lactobacillales, *Enterobacter*, *Klebsiella*, Enterobacteriaceae, Bacilli, Clostridiaceae, Enterobacteriales, Bacillales, Enterococcaceae, *Enterococcus*, and *Streptococcus* (*CER* = 0.41, full list of VIPs in Supplementary Table 3). Univariate differential abundance analysis was also performed via ANCOM-BC2 correcting for delivery mode and antibiotic exposure (Supplementary Table 4). This also confirmed that infants in the Test group presented lower levels of *Enterobacter* and *Klebsiella* and higher levels of Lachnospiraceae and Ruminococcaceae compared to Control. Finally, at 12 months of life, no overall differences were observed between the two groups.

Overall, the pairwise comparisons highlighted that infants from the Test group were associated with the presence of Erysipelotrichaceae, Erysipelotrichales, and Erysipelotrichia compared to both groups (Reference and Control) and with Ruminococcaceae and Lachnospiraceae compared to Control infants. Of interest, the Control group presented higher *Enterobacter* and *Klebsiella* genera compared to the Test group (Fig. 1E). The ratio obtained from OTUs detected to differentiate Test from Control significantly separated the two groups and was not influenced by sex, delivery mode, and antibiotic exposure (Fig. 1F). Reference infants presented more Pasteurellales, Pasteurellaceae, *Haemophilus*, Veillonellaceae, and *Veillonella* (Fig. 1G) compared to both formula-fed groups.

### Intake of test formula modulates the infant fecal metabolome

**Fig. 2:**
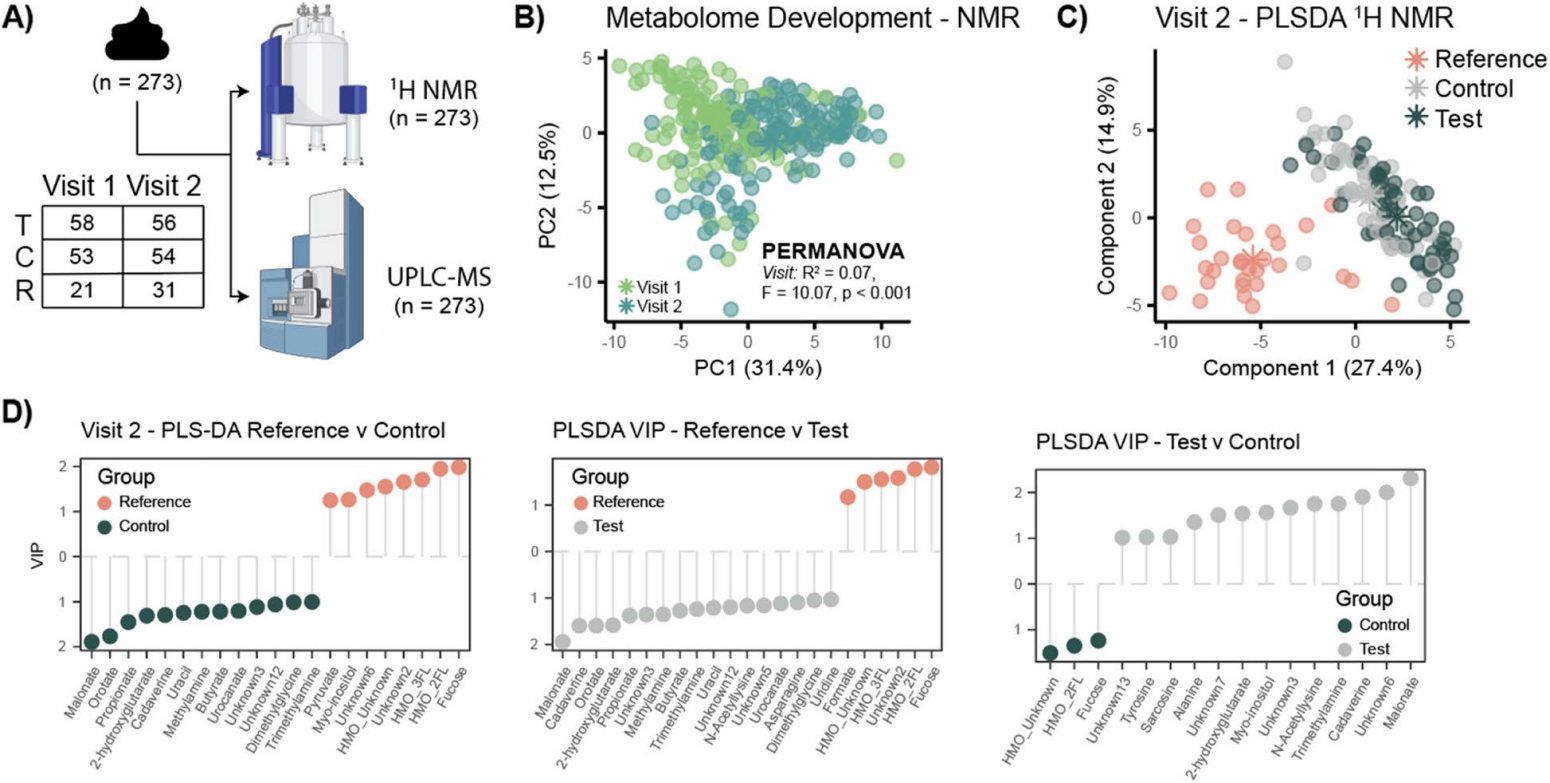
Test infant formula influences fecal metabolic profiles at 3 months of age. **A** Available fecal samples (*n* = 273) collected from infants at enrollment (Visit 1 and Visit 2) were analyzed via ^1^H NMR spectroscopy and UPLC-MS. **B** A time-dependent fecal metabolome development was observed (PERMANOVA, R2 = 0.07, F = 10.07, *p* < 0.001). **C** Fecal metabolic profiles differed between groups at Visit 2. **D** Pairwise PLS-DA models of the log-transformed integrated peak areas were constructed to investigate metabolites driving group differences at 3 months. VIP plots comparing Reference versus Control (CER 0.06), Reference versus Test (CER 0.02), and Test versus Control (CER 0.23) highlighted key differences. Reference infants excreted greater amounts of human milk oligosaccharides compared to the formula-fed groups, which presented greater amounts of malonate, orotate, cadaverine, and uracil

The metabolomic and lipidomic profiles of stool samples from enrollment (*n* = 132) and 3 months of life (*n* = 141) were measured using ^1^H nuclear magnetic resonance (NMR) spectroscopy and ultra-performance liquid chromatography-mass spectrometry (UPLC-MS) via Biocrates MxP® Quant 500 kit (Fig. 2A).

As observed for fecal microbiome, a clear age-dependent development was observed for the fecal metabolome (Fig. 2B). Features driving the separation included human milk oligosaccharides (HMOs), 2′fucosyllactose (2′FL), and 3′fucosyllactose (3′FL), which were enriched in early timepoints, and alanine, cadaverine, tyrosine, methionine, and fumarate, which were enriched in later timepoints. Pairwise PLS-DA models were built on the NMR profiles. At enrollment, no differences in the fecal biochemical profiles were observed between the groups (*CER* > 0.5), while at 3 months differences were observed (Fig. 2C). Pairwise PLS-DA models were built between the groups, and features driving the separation were extracted (Fig. 2D). As expected, stools from Reference infants contained higher levels of fucose and the human milk oligosaccharides (HMOs), 2′fucosyllactose (2′FL), and 3′fucosyllactose (3′FL) compared to both Control and Test since the infants were breast-fed. Focusing on differences between Test and Control infants (*PLS-DA CER* = 0.23) highlighted that Test infants excreted greater amounts of malonate, cadaverine, sarcosine, trimethylamine, *N*-acetyllysine, *myo*-inositol, 2-hydroxyglutarate, alanine, and tyrosine compared to the Control group. Compared to Reference infants, the Test group excreted greater butyrate, propionate, methylamine, trimethylamine, dimethylglycine, *N*-acetyllysine, asparagine, malonate, cadaverine, 2-hydroxyglutarate, orotate, urocanate, uracil, and uridine (*PLS-DA CER* = 0.02). Similarly, the Control group excreted greater amounts of malonate, butyrate, propionate, 2-hydroxyglutarate, cadaverine, uracil, methylamine, orotate, urocanate, dimethylglycine, and trimethylamine compared to the Reference infants (*PLS-DA CER* = 0.06).

**Fig. 3:**
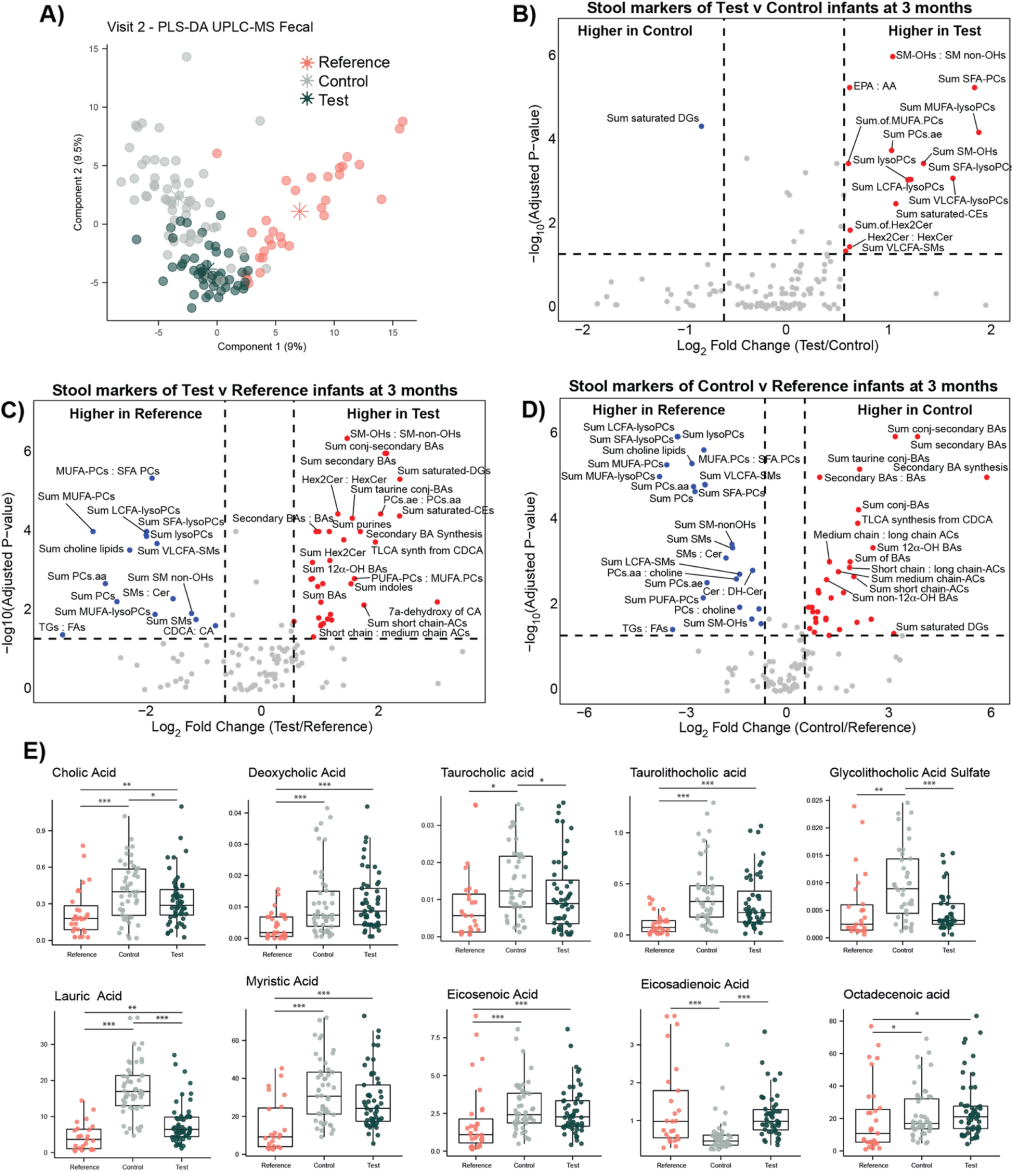
Variation in fecal metabolites and functions across the study groups at 3 months of age measured by UPLC-MS. **A** PLS-DA scores plot comparing fecal metabolic phenotypes of Reference, Control, and Test infants. Volcano plots comparing the metabolism indicators measures from **B** Test vs Control, **C** Test vs Reference, and **D** Control vs Reference. Highlighted indicators have an absolute fold change value > 1.5. Wilcoxon rank-sum test was used to detect significant differences between the groups, and *p*-values were corrected using Benjamini-Hochberg (*p-adjusted* < 0.05). **E** Fecal bile acids and fatty acids identified to show a trend for being consistent between Reference and Test infants but different from Control infants. Statistical significance determined by Kruskal–Wallis tests followed by pairwise Wilcoxon tests corrected using Benjamini-Hochberg. Significance: *p < 0.05, **p < 0.01, ***p < 0.001

A semi-targeted UPLC-MS method was also used to broadly characterize the fecal metabolomes and lipidomes of the study infants. At 3 months, clear metabolic variation between the groups was observed (*PLS-DA CER* = 0.09, Fig. 3A). Differences between the Test and Control group predominantly included alterations in lipid excretion, with the Test infants excreting higher amounts of different acylcarnitines, various ceramide-related species, phosphatidylcholines, such as PC aa C38:0, and cholesteryl esters, like CE (15:0). Control infants excreted higher amounts of lactate, symmetric dimethylarginine (SDMA), asymmetric dimethylarginine (ADMA) and the fatty acids, lauric acid, myristic acid, palmitic acid, and the bile acids cholic acid and glycolithocholic acid-sulfate (Supplementary Table 6).

To assess the fecal metabolic differences that were directly related to variation in the chemical content of the Test and Control milks, the same UPLC-MS profiling was performed on the infant formulas (Supplementary Fig. 3). Test milk contained greater amounts of several ceramides, dihydroceramides, glycosylceramides, phosphatidylcholines (including PC aa C38:0), sphingomyelins, and the cholesteryl ester CE (15:0) compared to the Control milk and lower amounts of choline, trigonelline, glycine, and proline. As such, increased excretion of ceramides and phosphatidylcholines are likely explained by compositional differences in the milk formulas. However, no differences were observed in the fatty acids, except for myristic acid (14:0) and octadecenoic acid (18:1) being higher in the Test formula. The same approach has been performed on breast milk samples, where 424 of 630 metabolites measured were quantified [23].

A range of metabolism indicators were calculated from the fecal metabolic profiles, with several significantly differentiating the two groups. Using a significance threshold of 1.5-fold higher/lower in Test versus Control and an FDR-adjusted *p* < 0.05, stools from Test infants contained a greater sum of various lysophosphatidylcholines, hydroxylated sphingomyelins, and saturated cholesteryl esters compared to Controls (Fig. 3B; Supplementary Table 7). Metabolites and lipids commonly differentiating Control from Test and Reference groups are reported in Supplementary Table 8. This included a range of ceramides, sphingomyelins, acylcarnitines, and fatty acids. Univariate analysis confirmed that the Control group excreted higher amounts of cholic acid, taurocholic acid, and glycolithocholic acid sulfate compared to both Reference and Test (Fig. 3E). Interestingly, cholic acid and taurocholic acid were modestly higher in the Test formula compared to the Control formula. Breast-fed infants excreted greater amounts of deoxycholic acid and taurolithocholic acid compared to both Control and Test. Differences were also confirmed in fatty acid profiles with lauric acid (also known as dodecanoic acid; 12:0) and eicosadienoic acid (20:2) being respectively higher and lower in Control infants compared to both Reference and Test. Reference stools contained lower amounts of myristic acid (tetradecanoic acid), eicosenoic acid, and octadecenoic acid compared to both Control and Test stools.

In contrast to the Reference group, the Test infants excreted higher amounts of ceramide-related compounds, glutamate, hydroxyglutaric acid, PAG, xanthine, hypoxanthine, spermidine, taurolithocholic acid, and hydroxyhexadecenoylcarnitine. Reference infants excreted greater amounts of homocysteine, ceramide-related compounds, and two sphingomyelins (Supplementary Table 9). The metabolism indicators showed that Test had a greater sum of secondary bile acids, conjugated secondary bile acids, taurine-conjugated bile acids, and glycine-conjugated bile acids as well as higher 7a-dehydroxylation of cholic acid (Fig. 3C; Supplementary Table 10). Interestingly, microbial indole synthesis and the sum of indoles were greater in the Test group compared to Reference, which was not observed in the Control group. The Reference stools contained a greater sum of various lysophosphatidylcholines, phosphatidylcholines, and sphingomyelins and a greater ratio of sphingomyelins to ceramides and triglycerides to fatty acids.

Comparing Control and Reference groups, the formula-fed infants excreted greater ornithine, glutamate, acetylornithine, malonate, 3-hydroxyglutaric acid, spermidine, xanthine, hypoxanthine, the fatty acid, lauric acid, several ceramides-related molecules, carnitines, the bile acids, taurolithocholic acid, cholic acid, the microbial-derived phenylacetylglycine (PAG), and lysine, which can be dietary or microbial derived. The breast-fed infants excreted higher amounts of choline, lysophosphatidylcholines, ceramide-related compounds, and sphingomyelins (Supplementary Table 11). Comparing the metabolism indicators across these groups identified that Control stools contained a greater sum of bile acids, conjugated bile acids, secondary bile acids, conjugated secondary bile acids, taurine-conjugated bile acids (Fig. 3D; Supplementary Table 12). In addition, the sum of acylcarnitines, short-chain acylcarnitines, saturated fatty acid (SFA)-acylcarnitines, and mono-unsaturated fatty acids (MUFA)-acylcarinitines and the ratio of short-chain to long-chain acylcarnitines and medium-chain to long-chain acylcarnitines were also higher in Control. A greater sum of phosphatidylcholines (PCs), SFA-PCs, MUFA-PCs, lysoPCs, long-chain fatty acid-lysoPCs, and SFA-lysoPCs were noted in the stool of breast-fed infants, as well as a higher ratio of triglycerides to fatty acids.

### Milk feeding type modulates host plasma metabolome

**Fig. 4:**
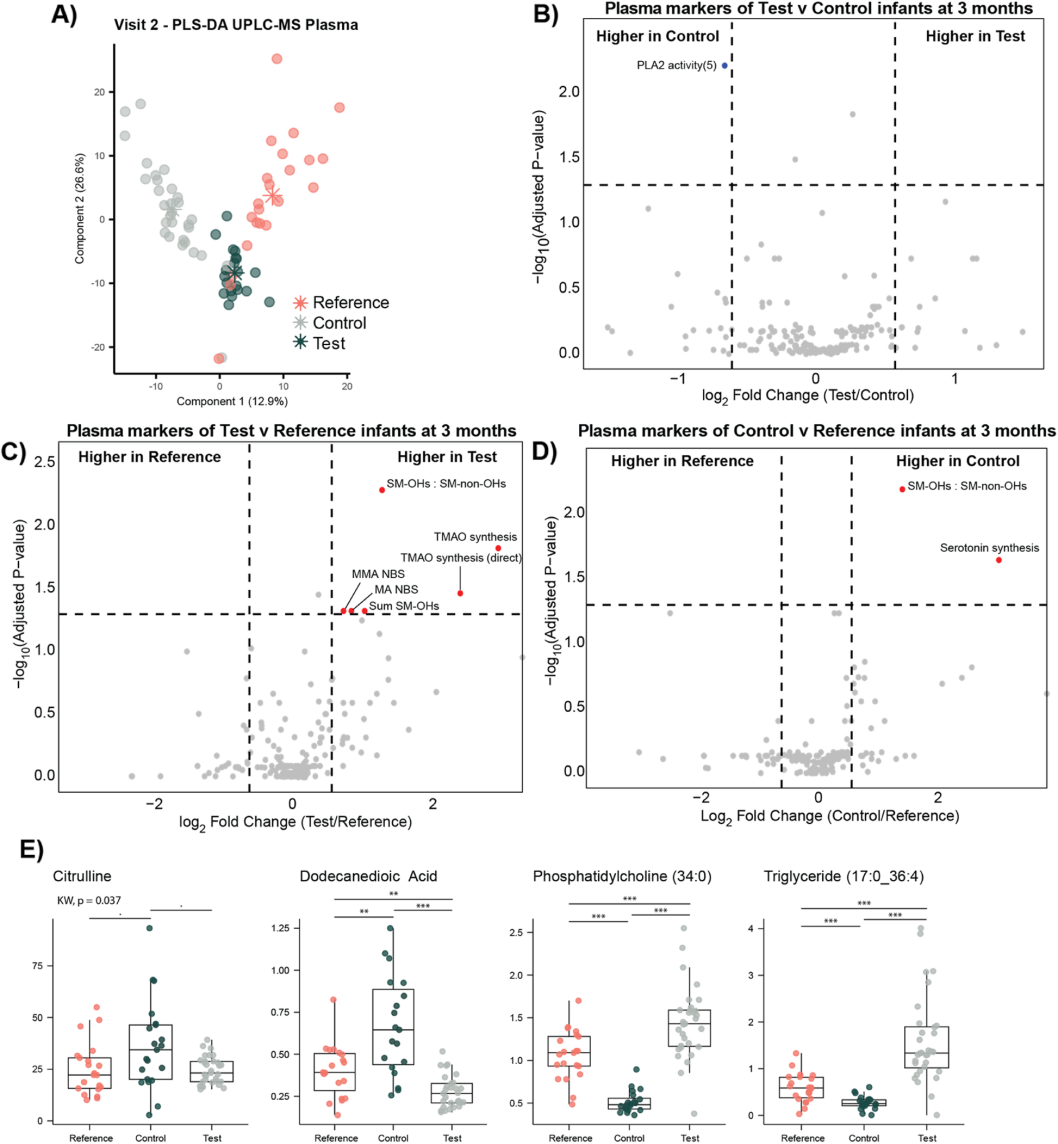
Variation in plasma metabolites and functions across the study groups at 3 months of age measured by UPLC-MS. **A** PLS-DA scores plot comparing the plasma metabolic phenotypes of Reference, Control, and Test infants. Volcano plots comparing the metabolism indicator measures from (**B**) Control vs Test, (**C**) Test vs Reference, and (**D**) Control vs Reference. Wilcoxon rank-sum test was used to calculate* p*-values, which were corrected using Benjamini-Hochberg (*p*-adjusted < 0.05). An absolute fold change threshold of 1.5 was applied. **E** Features identified to be consistent between Reference and Test infants but differ from Control infants. Kruskal–Wallis tests followed by pairwise Wilcoxon tests corrected using Benjamini-Hochberg. Significance: *p < 0.05, **p < 0.01, ***p < 0.001)

Semi-targeted UPLC-MS metabolomics was also performed on a total of 72 plasma samples (30 Test, 20 Control, and 22 Reference) collected at 3 months of age from the infants. A PLS-DA model constructed on these profiles revealed a clear separation between the three groups (*CER* = 0.07, Fig. 4A). A range of plasma metabolites and lipids were observed to differ between Test and Control infants (*PLS-DA CER* = 0.02). Test infants had greater circulating amounts of the microbial-host co-metabolites, PAG, and hippuric acid, as well as several ceramides, hexosylceramides, 26 phosphatidylcholines, and 62 triglycerides (Supplementary Table 13). Control infants had greater amounts of aspartate, glycine, serine, anserine, and citrulline in their circulation as well as dodecanedioic acid, dodecenoylcarnitine, octadecenoylcarnitine, octadecadienoic acid, and several ceramides, hexosylceramides, and phosphatidylcholines. Interestingly, 32 triglycerides and 10 phosphatidylcholines were commonly higher in both Reference and Test infants compared to the Control infants (Supplementary Table 14). Plasma citrulline and dodecanedioic acid were higher in Control compared to both the Reference and Test groups.

In contrast to the Reference group, the Test group had higher circulating amounts of isoleucine, leucine, valine, lysine, methionine, phenylalanine, *α*-aminobutyric acid, *α*-aminoadipic acid, hippuric acid, propionylcarnitine and 7 different ceramides, 4 hexosylceramides, 20 phosphatidylcholines and 3 lysophosphatidylcholines, 6 sphingomyelins, and 29 triglycerides (PLS-DA CER 0.06). Conversely, the Reference group had greater circulating amounts of cholic acid, dodecanedioic acid, homocysteine, tetradecenoylcarnitine, 12 ceramides, 7 phosphatidylcholines, 18 hexosylceramides, 6 sphingomyelins, and 40 triglycerides compared to Test (Supplementary Table 15).

A pairwise PLS-DA model showed that infants from the Control group had higher circulating amino acids and related metabolites compared to Reference infants, specifically isoleucine, leucine, valine, lysine, methionine, phenylalanine, threonine, *α*-aminobutyric acid, *α*-aminoadipic acid, methionine sulfoxide, and citrulline (PLS-DA CER 0.05). Propionylcarnitine, dodecanedioic acid, *p*-cresol sulfate, indole, choline, serotonin, cortisol, and dehydroepiandrosterone sulfate (DHEAS) were also higher in Control plasma compared to the Reference group. Conversely, the Reference group had higher amounts of circulating triglycerides (*n* = 84), hexosylceramides (*n* = 16), phosphatidylcholines (*n* = 16), ceramides (*n* = 6), sphingomyelins (*n* = 5), homocysteine, 3-methylhistidine, and the primary unconjugated bile acids, cholic acid, and chenodeoxycholic acid (a complete list can be found in Supplementary Table 16).

Analysis of the plasma metabolism indicators identified that infants receiving the Test formula had significantly lower PLA_2_ activity compared to Control infants (Fig. 4b; Supplementary Table 17) and significantly higher TMAO synthesis compared to the Reference infants (Fig. 4c; Supplementary Table 18). The ratio of hydroxylated-sphingomyelins to non-hydroxylated sphingomyelins was observed to be higher in both Control and Test groups compared to Reference, while the sum of hydroxylated sphingomyelins was only significantly higher in the Test group compared to the Reference individuals. Serotonin synthesis was found to be eightfold higher in the Control group compared to Reference (Fig. 4d; Supplementary Table 19).

### Type of milk feeding modulates the composition and functionality of the infant microbiota

**Fig. 5:**
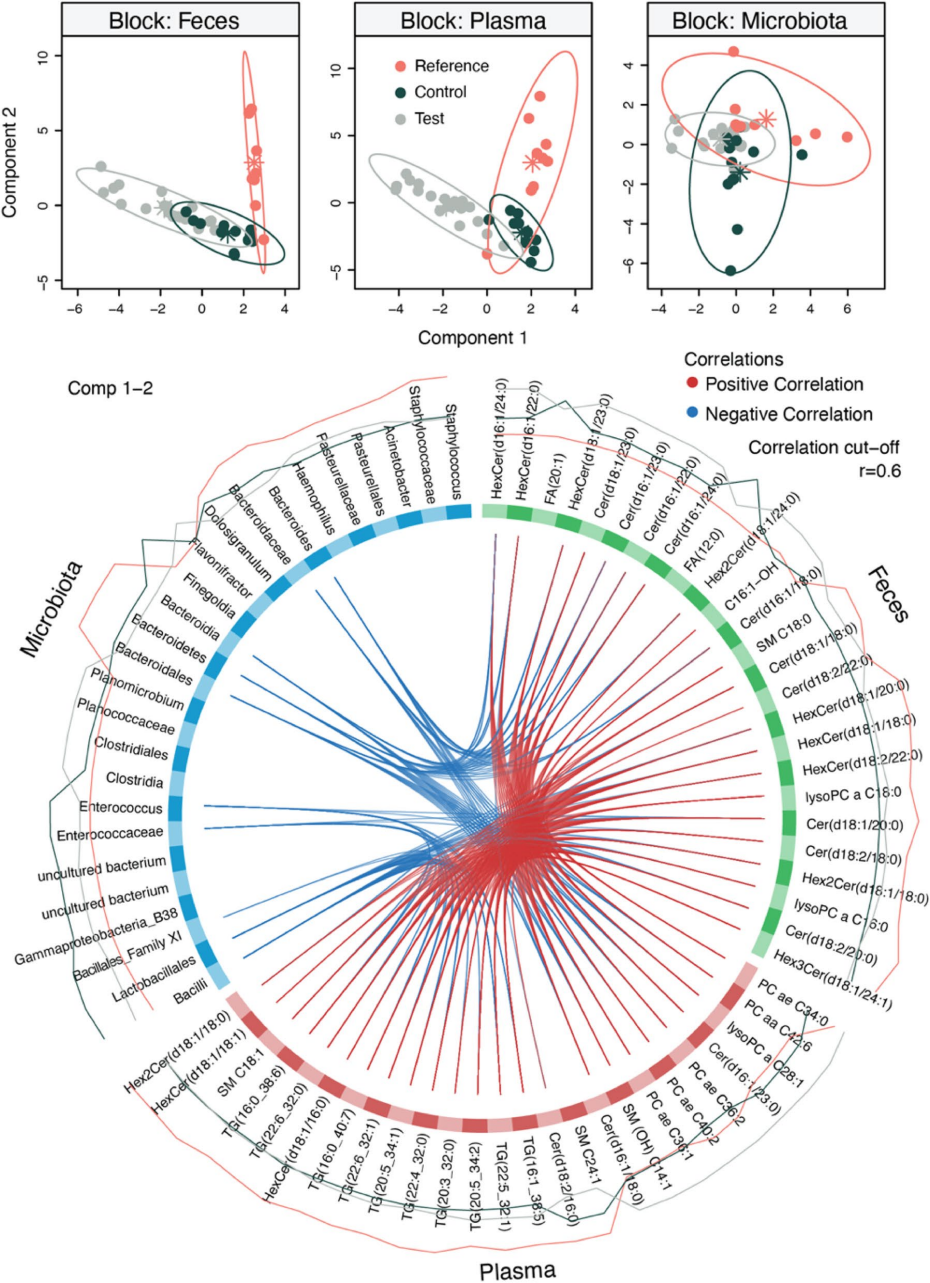
Multi-omics data integration of biosample collected at 3 months of life. The fecal microbiota, fecal metabolomic, and plasma metabolomic profiles measured at 3 months were integrated using DIABLO. Good classification performance was obtained (classification error rate = 0.20), and it was possible to observe that infants from the Reference group were clustering apart from the Test and Control groups. Circos plot highlighted correlation (*r* > 0.6) between variables from the different omics blocks. Enterococcaceae and *Enterococcus*, which were most abundant in Control infants, negatively correlated with plasma triglycerides (TG) and fecal ceramides (Cer), while Bacteroida, Bacteroidales, Bacteroidaceae, and *Bacteroides*, which were most abundant in Reference infants, negatively correlated with several fecal hexosylceramides (HexCer) and Cer and plasma phosphatidylcholines (PC). FA, fatty acids; Hex2 Cer, dihexosylceramides; lysoPC, lysophosphatidylcholines; SM, sphingomyelins

Omics data collected at 3 months of life was integrated as it presented the highest amount of paired acquired biological data (41 subjects with gut microbial profiles and plasma and fecal metabolomes). Projections recapitulated clustering observed in the single omics PLS-DA models, and the overall classification error rate of DIABLO was 0.20 (Fig. 5). Samples from the Reference group clearly separated from Test and Control groups in the two metabolomics blocks. The circos plot identified strong correlations between fecal and plasma metabolites. Enterococcaceae and *Enterococcus*, which were more abundant in the Control group, were negatively correlated with several plasma triglycerides and fecal ceramides. Bacteroidota, Bacteroidales, Bacteroidaceae, and *Bacteroides*, which were more abundant in the Reference group, were negatively correlated with several phosphatidylcholines, ceramides, and sphingomyelins present in plasma and stool.

### Weight gain, BMI, and total subcutaneous fat acquisition at 1 year of age

**Fig. 6:**
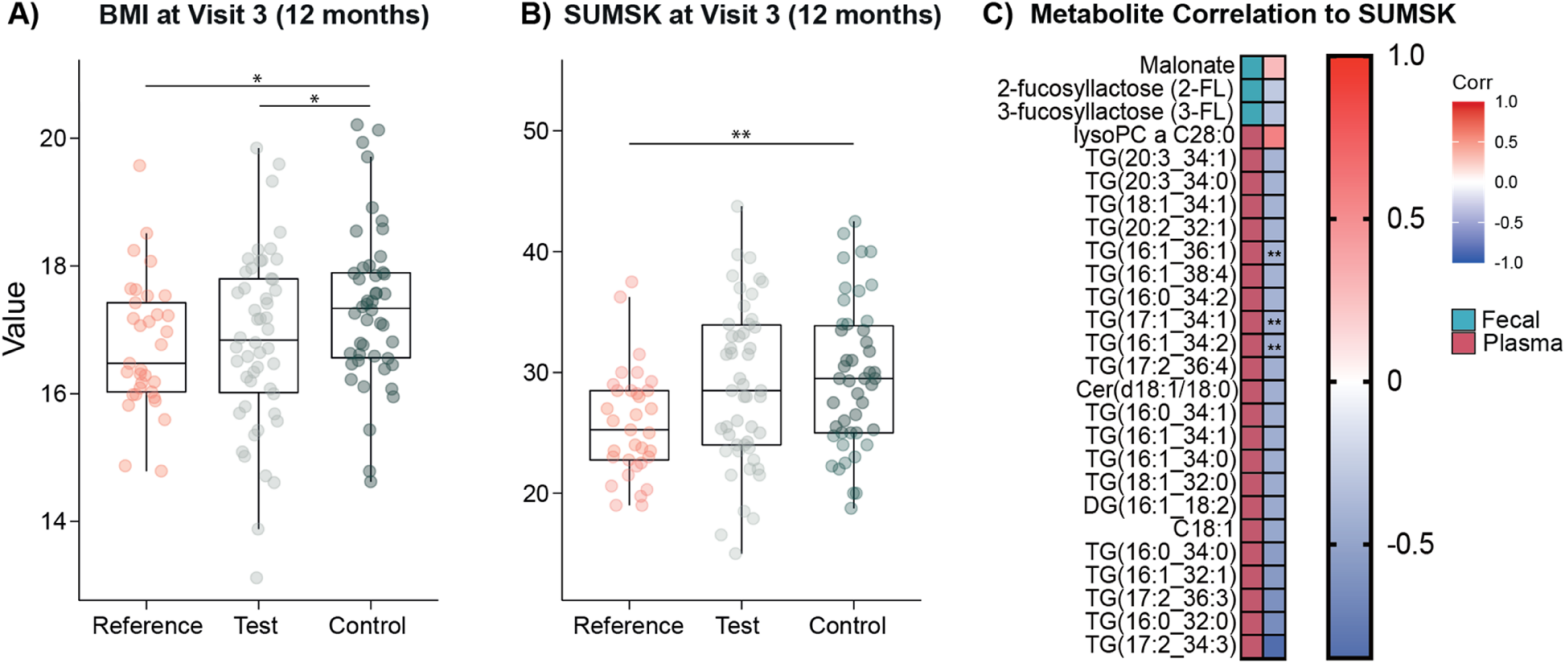
Differences in anthropometric measures at 12 months of life. **A** BMI was significantly higher in the infants receiving the control formula compared to the breast-fed Reference group and the infants receiving the novel test formula. **B** Subcutaneous fat, measured by the sum of skin thickness (SUMSK), was significantly higher in control compared to reference. Test group did not significantly differ from the other two groups. One outlier for sum of skin thickness (value = 52.5) was removed from the test group following Grubbs’ test (*p* = 0.005). Boxplots represent lower, middle (median), and upper quartile. ANOVA is followed by Tukey’s HSD test to calculate *p*-values. *p < 0.05, **p < 0.01. **C** Pearson correlations between SUMSK measured at 12 months and the fecal and plasma metabolites measured at 3 months. Correlations shown for significant features after a Benjamini-Hochberg correction (*p* < 0.1)

Anthropometric data was analyzed from the subset of study infants with matching microbial and metabolomic data (data from the full study cohort has been published [24]). At birth, all study groups had comparable anthropometric measures and gestational ages (ANOVA *p* > 0.05; Supplementary Fig. 1 A). At enrollment, the Reference group had greater BMI and weight-for-length Z-scores (WLZ) compared to the other study groups due to the significant difference in age (mean age in days: Reference, 18.3 ± 11.1; Test, 11.5 ± 9.86; Control, 9.86 ± 9.40; Supplementary Fig. 1B). At 3 months, no differences in the anthropometric measures were observed between the study groups. No group differences were noted after stratifying the infants by delivery mode or sex. By 1 year of age, infants from the Control group had significantly higher BMI scores (mean 17.37 ± 1.29) compared to both the Test (mean 16.76 ± 1.35) and the Reference (mean 16.66 ± 1.04) groups (ANOVA followed by Tukey’s HSD test; Fig. 6A). Significance was retained after correcting for sex, weight at birth, gestational age, delivery mode, and antibiotics exposure via linear regression. Total subcutaneous fat, measured by skin fold thickness (SUMSK), was also significantly higher in Control infants (mean 29.52 ± 6.02) compared to Reference infants (mean 26.07 ± 4.60; *p* = 0.01; Fig. 6B). Interestingly, the Test group did not have significantly different SUMSK compared to either Reference or Control (mean 28.58 ± 6.47).

Finally, correlations of the different omics blocks at 3 months of age to infant BMI and total SUMSK measured at 1 year of life were investigated. This was performed on each block using associated metadata (108 fecal NMR profiles, 103 fecal and 58 plasma mass spectral profiles, 117 microbial profiles). As DIABLO only permits classification with a discrete response vector, Pearson correlations between fecal and plasma metabolites and the phenotypic outputs were calculated. Conversely, for the microbiota data, Spearman correlations were used. Obtained *p*-values were adjusted using Benjamini–Hochberg correction, and adjusted *p* < 0.1 were considered significant (Supplementary Table 20). Fecal NMR data highlighted the HMOs, 2′FL, and 3′FL were negatively correlated with SUMSK, while malonate was positively correlated with this measure. Conversely, no fecal metabolic features acquired through mass spectrometry correlated with SUMSK. Plasma mass spectrometry data showed a positive correlation of lysoPC a_C28:0 (*R* = 0.57) with SUMSK, while 19 different triglycerides were negatively correlated with the same measurement including TG(16:1_34:2), TG(16:1_36:1), and TG(17:1_34:1), which were increased in the Test infant plasma compared to the Control group. The plasma triglyceride TG(17:2; 34:3) was strongly negatively correlated with SUMSK (*R* = − 0.85). No significant correlations were observed between microbial taxa and BMI or SUMSK following Benjamini–Hochberg correction.

## Discussion

In this study, the presence of large, milk phospholipid-coated lipid droplets in infant formula modified the composition and metabolic function of the developing infant gut microbiota and its biochemical interactions with the host. In addition, this novel Test formula milk prevented the significant increase in body fat and BMI gain seen in standard formula-fed infants compared to those receiving breast milk. Breastfeeding is consistently associated with a long-term protective role in obesity, cardiovascular disease, and hypertension; however, the mechanisms underlying this protection are poorly understood [11]. These findings suggest that the lipid component of human milk may contribute to this protection, partially mediated through the shaping of the maturing microbiota and their role in holobiont metabolism and physiology.

The establishment of the fecal microbiota in vaginally born and breast-fed infants during the first months and years of life has been well characterized. A high abundance of *Bifidobacterium* and/or *Bacteroides* is typically observed in the first days of life, which further increases during exclusive breastfeeding until 4-month postpartum when their abundance (especially *Bifidobacterium*) progressively declines until 24 months of age. From birth, there is also a steady decline in the abundance of Enterobacteriaceae, Streptococcaceae, and Clostridiaceae with a simultaneous increase in Lachnospiraceae and Ruminococcaceae [25]*.* In this study, infants receiving the Test formula harbored less *Enterobacter*, *Klebsiella*, *Enterococcus*, *Streptococcus*, Enterobacteriaceae, Clostridiaceae, and Enterococcaceae at 3 months compared to those receiving standard cow milk-based formula with prebiotics and a greater abundance of Ruminococcaceae and Lachnospiraceae. Previous in vitro and preclinical studies have shown that milk fat globule membranes increase the abundance of butyrate producers, with one study identifying that glycoproteins in the membrane could prevent adhesion of the colonic microbiota and increase butyrate production [71, 72]. These findings indicate that the large milk phospholipid-coated lipid droplets in the Test formula resulted in gut microbiota maturation trajectories more like that of healthy breast-fed infants.

Previous studies demonstrated a reduction in pathobionts following intake of infant formula with prebiotics through the stimulation of bifidobacteria [26]. Several taxa that were noted to be less abundant in the Test group infants compared to the Control group infants at 3 months include bacterial genera that have been described as risk factors for infections, including members of the ESKAPE group of pathogens (e.g., *Enterobacter*). This indicates that the large milk phospholipid droplets may further enhance the restriction of pathobionts seen with infant formula containing prebiotics. This is consistent with previous infant and adult studies showing that MFGM supplementation can reduce the risk of infections, such as gastrointestinal infections [4, 5, 7, 27]. This suggests that lipids in the Test infant formula droplets, or lipid changes induced by these lipid droplets, may confer colonization resistance to the infants. Lipid species are gaining increasing attention for their antimicrobial potential [28, 29]. These species predominantly include fatty acids and monoglycerides, which can disrupt bacterial cell membranes [30]. The antimicrobial properties of fatty acids are influenced by their structural characteristics, including chain length and degree of unsaturation. For example, fatty acids greater than 12 carbons in length are effective against gram-positive bacteria, while those 6 carbons or lower are effective against gram-negative bacteria. Similarly, unsaturated fatty acids are typically more efficacious against gram-positive bacteria. The fecal abundance of several fatty acids was observed to differ across the groups, which may contribute to the variation in gut microbial populations between breast-fed and formula-fed infants. For example, lauric acid (C12:0), which has been shown to be an effective inhibitor of *Propionibacterium acnes*, was significantly higher in the Control stools compared to the Reference group, but did not differ between the Reference and Test infants. Conversely, both the Reference and Test stools contained greater amounts of eicosadienoic acid (20:2) compared the Control infants. In addition, glycosylated proteins present at the surface of the lipid droplet can also provide binding sites to these bacteria, like what is observed for HMOs [31, 32], preventing the adhesion of pathogens to intestinal epithelial cells.

This compositional variation between Test and Control infants was accompanied by differences in several fecal and plasma metabolites at 3 months related to gut microbial metabolism. This included a higher amount of fecal trimethylamine, a microbial breakdown product of choline, and plasma PAG and hippurate in Test infants compared to the Control infants. Both PAG and hippurate are microbial-host co-metabolites derived from microbial phenylalanine degradation, with hippurate also derived from the microbial processing of polyphenols. Hippurate has been highlighted as marker of gut microbial diversity and, consistent with the lower BMI of the Test infants, has been inversely associated with blood pressure and metabolic syndrome in adults [33, 34]. Fecal tyrosine and histidine, which are typically degraded by the microbiota to *p*-cresol and imidazole-propionate respectively, were higher in the Test group infants. Given the matched amino acid content of the two formulas, this indicates that the bacterial degradation of these amino acids is lower following the intake of these lipid droplets. This is noteworthy given the adverse health effects attributed to the bacterial products of tyrosine and histidine metabolism including type 2 diabetes [35] and cardiovascular diseases [36, 37]. Lastly, Test group infants had lower circulating amounts of GABA, aspartate, and serine compared to the Controls, which can arise from microbial or host metabolism. GABA and the D-forms of aspartate and serine have been implicated in the microbiota-gut-brain axis with potential to modulate neurodevelopment [38–40]. Interestingly, serotonin synthesis (based on plasma serotonin and tryptophan measures) was observed to be eightfold higher in Control versus Reference individuals but was not found to differ between Test and Reference infants. This is consistent with Control infant formula containing notably higher amounts of tryptophan compared to the Test formula.

Formula milk consumption has been associated with rapid weight gain [41] and is estimated to increase the odds of developing obesity later in life [12]. Consistently, the Control formula-fed infants in this study had significantly greater BMI measures at 1 year of life compared to those receiving breast milk. The presence of large, milk phospholipid-coated lipid droplets containing a mixture of dairy and vegetable lipids in the formula milk attenuated this weight gain and adiposity in the Test group infants. This protective effect could be attributed to their influence on the developing metabolic state of the infant and their lipid and energy processing capacity, and/or it could be related to induced shifts in microbiota maturation and their role in holobiont metabolism. For example, several of the taxa found to be less abundant in the Test group infants compared to the Controls at 3 months have been implicated as risk factors in noncommunicable diseases. *Klebsiella* has been linked with obesity in both adults and children [42, 43], and different *Enterobacter* and *Streptococcus* species [44] have been associated with infants at high risk of developing obesity. Moreover, *Streptococcus* abundance at 3 months of age has been positively connected to a higher BMI in a cohort of children aged 5 years [45]. Furthermore, *Enterobacter cloacae*, isolated from obese humans, has been shown to induce inflammation and increase subcutaneous fat accumulation in murine models [46, 47].

In terms of metabolic priming of the host, breast-fed infants have been previously reported to have higher circulating concentrations of plasma triglycerides compared to formula-fed infants [48–50]. This is hypothesized to reflect greater mobilization of body fat from adipose tissue for the generation of energy to support infant growth, particularly the brain. Several plasma lipids, including triglycerides, phosphatidylcholines, and sphingomyelins, were commonly more abundant in the breast-fed infants and those receiving the Test formula compared to the Control group. Interestingly, 19 circulating triglycerides, a diglyceride and an acylcarnitine, were found to be negatively correlated with the total sum skin thickness, supporting the link between enhanced mobilization and metabolism of triglycerides and reduced body fat. In addition, the higher triglycerides in the Reference plasma may reflect the greater diversity of these lipids in breast milk compared to formula milk [51, 52]. In this context, the presence of large, milk phospholipid-coated lipid droplets containing a dairy and vegetable lipid mixture appears to reduce this variation. Conversely, greater triglyceride storage could be occurring in the Control group infants. Formula-fed infants have a greater proportion of white adipose tissue (WAT), specialized for energy storage, while breast-fed infants possess greater amounts of mitochondria-rich brown (BAT) and beige adipose tissue (BeAT), optimized for energy expenditure and thermogenesis. Breast milk components, such as alkylglycerols and metabolites of linoleic acid, have been proposed to drive these developmental differences, preventing the transformation of BeAT into WAT [53, 54]. Given the differences in stool and plasma triglycerides and fatty acids, coupled with variation in BMI and adiposity, the influence of the Test formula lipid droplets on adipose tissue phenotypes warrants further investigation.

Breast milk also contains higher amounts of cholesterol than formula milk, which could downregulate hepatic hydroxymethylglutaryl coenzyme A (HMG-CoA) activity decreasing de novo cholesterol synthesis [55]. Indeed, the sum of various conjugated and secondary bile acids was higher in stools from Control and Test group infants. The plasma concentrations of chenodeoxycholic acid, a primary bile acid arising from bacterial deconjugation, was higher in the Reference group compared to the Control group but was comparable between the Reference and Test individuals. Chenodeoxycholic acid has been shown to have anti-obesogenic potential by signaling through TGR5 to promote whole-body energy expenditure in BAT [56–58] and has been observed to be negatively correlated with insulin sensitivity [59].

The presence of large, milk phospholipid-coated lipid droplets in infant formula may also modify the community structure of the microbiota through the provision of different substrates for the microbes. From this perspective, these lipid droplets and their constituent (phospho)lipids may represent a new class of microbial modulators. Data integration revealed a negative correlation between *Bacteroides* taxa and different lipid species, mainly ceramides and phosphatidylcholines, in both stool and plasma. This suggests that *Bacteroides* taxa could metabolize MFG-derived lipids reducing their bioavailability in the gut and those reaching the systemic circulation. Recent studies indicated that *Bacteroides* not only have the metabolic capability to digest dietary lipids but also have the anabolic capacity to synthesize functional lipids [60–63]. There is growing interest into the role of *Bacteroides* in early life with recent data suggesting that *Bacteroides* may reduce atopic eczema risk and influence cognitive development [64–66]. Studies have shown a higher prevalence of these bacteria in vaginally born and breast-fed infants compared to those born by C-section or before term [67, 68]. In agreement with these findings, a recent study reported the effect of bovine milk fat globule membrane in modulating *Bacteroides* in vaginally born infants [69].

The consumption of Test formula resulted in a greater abundance of Ruminococcaceae and Lachnospiraceae at 3 months of age, before the introduction of solid foods. These bacterial groups contain known butyrate producers. While no differences were noted in fecal butyrate at this sampling point, these observations indicate that the infant gut microbiota has been primed for butyrate production prior to weaning. This warrants further investigation given the inverse association between SCFA-producing bacteria in early life and future BMI in childhood [70]. Preclinical and clinical studies support the functionality of MFGM, especially MFGM glycoproteins in modulating butyrate producers [71, 72]. Oral butyrate supplementation has also been demonstrated to positively influence BMI, glucose metabolism, and inflammation in children with obesity [73].

The RCT design is a major strength of this study allowing the impact of MFMG on the infant holobiont and its phenotypic development to be investigated. All groups were balanced for sex, delivery mode, gestational age, birth weight, and antibiotic intake minimizing their influence on the observed differences. Moreover, the integrated application of microbial and metabolic profiling on fecal and plasma samples allows microbiota and host metabolism to be comprehensively assessed in response to different feeding approaches at the gut and systemic level. The sparse sampling time points (enrollment, 3 months, and 1 year) are a limitation of the current work preventing the dynamic maturation of the infant, its microbiota and metabolism, and their response to infant nutrition, from being studied at high resolution. Moreover, further work is required to understand the biomolecular mechanisms through which MFMGs shape gut microbial and holobiont development. While this was an exploratory study, future work incorporating larger sample sizes will provide greater statistical power allowing more subtle alterations induced by MFMGs on microbial-host interactions to be gleaned and their implications for human development to be studied.

## Conclusions

The presence of large milk phospholipid-coated lipid droplets in formula milk positively influences the development of the infant gut microbiota, their biochemical profile, and their body composition. This has potential to reduce colonization of the infant gut by potentially pathogenic bacteria and result in long-term health benefits, such as improved anthropometric trajectories and reduced risks of obesity and type- 2 diabetes mellitus development.

## Supplementary Information

Supplementary Material 1. Supplementary Fig. 1. Anthropometrics at birth and enrollment. Anthropometric measurements at a) birth and at b) enrollment. No differences between groups were observed at birth between the groups for any measures. At enrollment, Reference infants had greater measures for BMI, head circumference, length, weight, and weight-for-length (WFL). This was due to the older age of these infants at enrolment compared to the other two study groups. Boxplots represent first (lower), median and third (upper) quartile. ANOVA followed by Tukey’s HSD test. Significance * *p* < 0.05, ** *p* < 0.01. Supplementary Fig. 2. Fecal microbiota development and group differences. A) Operational taxonomic units (OTUs) that correlated with PC1 (|cor|> 0.6) highlighted Lachnospiraceae and Ruminococcaceae were associated with later timepoints while the earlier samples contained a greater abundance of Proteobacteria. B) Alpha diversity was calculated at each timepoint using three different metrics (Observed OTUs, Chao1 and Shannon). Groups were compared with the Kruskal–Wallis test. Significance was obtained at enrollment for both observed OTUs and Chao1 metrics and at 3 months only for observed OTUs. Following pairwise comparisons with Wilcoxon test and BH correction, the Reference group displayed a significantly higher alpha diversity compared to Control (Observed OTUs metric, *p* = 0.0056 and Chao1 metric, *p* = 0.0024) at enrollment. Differences between Test and Control groups were close to significance. At 3 months, Reference infants had a significantly lower alpha diversity compared to the Test group (*p* = 0.0016). The Control group had close to significant lower alpha diversity compared to the Test group (*p* = 0.072). No significant differences were observed using the Shannon diversity index. Boxplots represent lower, middle (median) and upper quartile. Significance: · *p* < 0.1, * *p* < 0.05, ** *p* < 0.01. C) Fecal microbial profile differences at the different sampling points. Groups were significantly different at each of the analyzed timepoints (PERMANOVA, *p* < 0.05), with maximum variance observed at 3 months of life, driven by the Reference group. Asterisks represent group centroids. Supplementary Table 1. Population characteristics of the analyzed samples from MERCURIUS Study. Study population characteristics for the infants with anthropometry, microbial and metabolic data. Values are reported as percentage or represent the mean of the group ± the standard deviation. Continuous variables were tested with ANOVA, while categorical data were tested pairwise with the Fisher’s exact test (ns = p > 0.05). Supplementary Table 2. VIPs of extracted OTUs influencing PLS-DA model (CER = 0.48) built on Test and Control groups at enrollment. Supplementary Table 3. VIPs of extracted OTUs influencing PLS-DA model (CER = 0.41) built on Test and Control groups at 3 months. Supplementary Table 4. ANCOM-BC2 differential abundance analysis. Supplementary Table 6. Extracted VIP score from pairwise PLS-DA model (CER = 0.08) comparing the Control and Test fecal metabolic profiles measured by UPLC-MS at 3 months of life. Supplementary Fig. 3: Metabolic and lipid content of the Test and Control infant formula measured by the liquid chromatography-mass spectrometry. Supplementary Table 7. Fecal metabolism indicators found to significantly differ between Test and Control infants. Wilcoxon rank-sum test was used to calculate the *p*-values, which were FDR corrected (q = 0.05). A fold change threshold of > 2 and < - 2 was applied. Supplementary Table 8. Fecal metabolites measured by UPLC-MS commonly different in Reference and Test infants compared to Control infants. Green shaded metabolites indicate metabolites higher in Reference and Test stools compared to Control stools and Orange shaded metabolites highlight those lower. Supplementary Table 9. Extracted VIP score from pairwise PLS-DA model (CER = 0.04) comparing the Test and Reference fecal metabolic profiles at 3 months of life measured by UPLC-MS. Supplementary Table 10. Fecal metabolism indicators found to significantly differ between Test and Reference infants. Wilcoxon rank-sum test was used to calculate the *p*-values, which were FDR corrected (q = 0.05). A fold change threshold of > 2 and < - 2 was applied. Supplementary Table 11. Extracted VIP score from pairwise PLS-DA model (CER = 0.05) comparing the Reference and Control fecal metabolic profiles at 3 months of life measured by UPLC-MS. Supplementary Table 12. Fecal metabolism indicators found to significantly differ between Control and Reference infants. Wilcoxon rank-sum test was used to calculate the *p*-values, which were FDR corrected (q = 0.05). A fold change threshold of > 2 and < - 2 was applied. Supplementary Table 13. Plasma metabolites measured by UPLC-MS observed to differ in the PLS-DA model between Test and Control infants at 3 months. Supplementary Table 14. Common metabolic variation in the plasma metabolites of Test and Reference infants compared to Control infants at 3 months. Metabolic differences determined by PLS-DA models (VIP > 1). Supplementary Table 15. Plasma metabolites measured by UPLC-MS observed to differ in the PLS-DA model (CER = 0.06) between Test and Reference infants at 3 months. Supplementary Table 16. Plasma metabolites measured by UPLC-MS observed to differ in the PLS-DA model (CER = 0.07) between Control and Reference infants at 3 months. Supplementary Table 17. Plasma metabolism indicators found to significantly differ between Control and Reference infants. Wilcoxon rank-sum test was used to calculate the *p*-values, which were FDR corrected (q = 0.05). A fold change threshold of > 1.5 and < - 1.5 was applied. Supplementary Table 18. Plasma metabolism indicators found to significantly differ between Test and Reference infants. Wilcoxon rank-sum test was used to calculate the *p*-values, which were FDR corrected (q = 0.05). A fold change threshold of > 1.5 and < - 1.5 was applied. Supplementary Table 19. Plasma metabolism indicators found to significantly differ between Control and Reference infants. Wilcoxon rank-sum test was used to calculate the *p*-values, which were FDR corrected (q = 0.05). A fold change threshold of > 1.5 and < - 1.5 was applied. Supplementary Table 20. Plasma and fecal metabolites measured at 3 months significantly correlated with total sum skin thickness measures at one year in all infants. Pearson correlations shown for all features significant after a Benjamini–Hochberg correction (p < 0.1).

## Data Availability

All data included in this manuscript will be deposited in MetaboLights (https://www.ebi.ac.uk/metabolights/) once accepted for publication.
