# Supplementary material for: Milk phospholipid-coated lipid droplets modulate the infant gut microbiota and metabolome influencing weight gain
Source: Microbiome. 2025 May 14;13:120. doi: 10.1186/s40168-025-02106-w (PMC12076826; doi:10.1186/s40168-025-02106-w)
Supplement: Supplementary file 2 — Supplementary Material 1. Supplementary Fig. 1. Anthropometrics at birth and enrollment. Anthropometric measurements at a) birth and at b) enrollment. No differences between groups were observed at birth between the groups for any measures. At enrollment, Reference infants had greater measures for BMI, head circumference, length, weight, and weight-for-length (WFL). This was due to the older age of these infants at enrolment compared to the other two study groups. Boxplots represent first (lower), median and third (upper) quartile. ANOVA followed by Tukey’s HSD test. Significance * p < 0.05, ** p < 0.01. Supplementary Fig. 2. Fecal microbiota development and group differences. A) Operational taxonomic units (OTUs) that correlated with PC1 (|cor|> 0.6) highlighted Lachnospiraceae and Ruminococcaceae were associated with later timepoints while the earlier samples contained a greater abundance of Proteobacteria. B) Alpha diversity was calculated at each timepoint using three different metrics (Observed OTUs, Chao1 and Shannon). Groups were compared with the Kruskal–Wallis test. Significance was obtained at enrollment for both observed OTUs and Chao1 metrics and at 3 months only for observed OTUs. Following pairwise comparisons with Wilcoxon test and BH correction, the Reference group displayed a significantly higher alpha diversity compared to Control (Observed OTUs metric, p = 0.0056 and Chao1 metric, p = 0.0024) at enrollment. Differences between Test and Control groups were close to significance. At 3 months, Reference infants had a significantly lower alpha diversity compared to the Test group (p = 0.0016). The Control group had close to significant lower alpha diversity compared to the Test group (p = 0.072). No significant differences were observed using the Shannon diversity index. Boxplots represent lower, middle (median) and upper quartile. Significance: · p < 0.1, * p < 0.05, ** p < 0.01. C) Fecal microbial profile differences at the different sampling points [file 40168_2025_2106_MOESM1_ESM.docx]

**Supplementary Information**


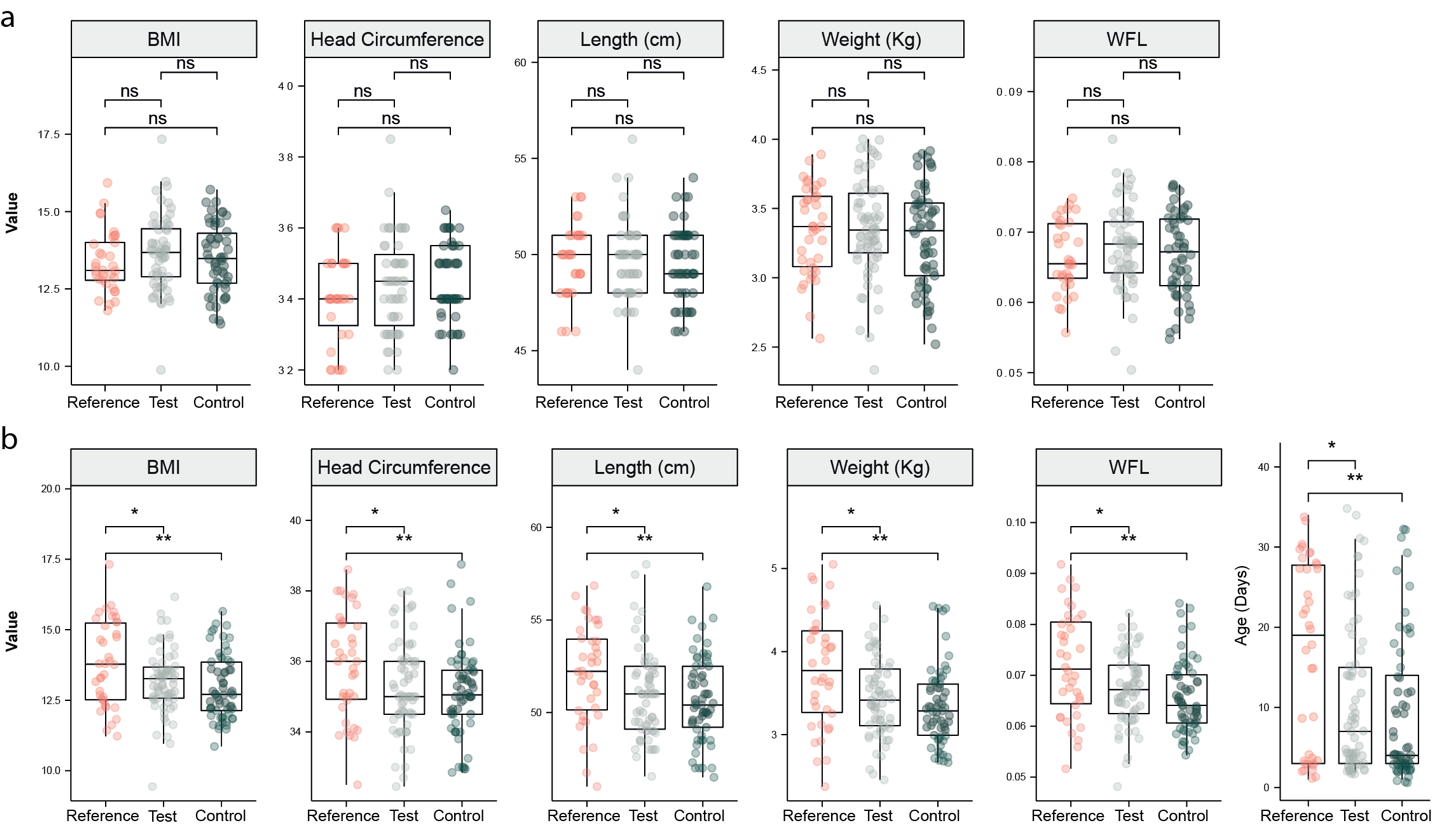


**Supplementary Figure 1.** Anthropometrics at birth and enrolment.

Anthropometric measurements at a) birth and at b) enrolment. No differences between groups were observed at birth between the groups for any measures. At enrolment, Reference infants had greater measures for BMI, head circumference, length, weight, and weight-for-length (WFL). This was due to the older age of these infants at enrolment compared to the other two study groups. Boxplots represent first (lower), median and third (upper) quartile. ANOVA followed by Tukey’s HSD test. Significance * p < 0.05, ** p < 0.01.

**
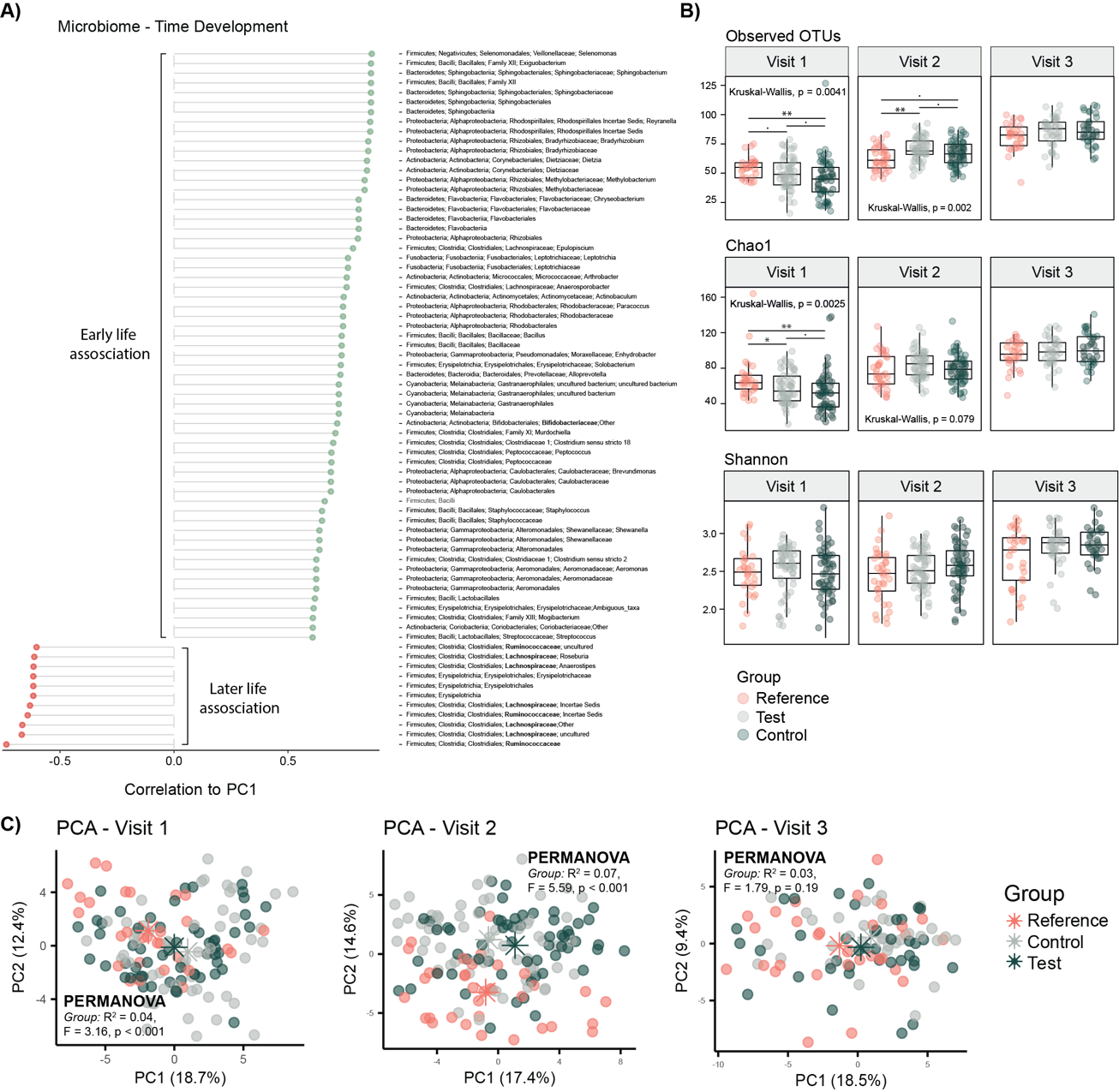
**

**Supplementary Figure 2. Fecal microbiota development and group differences.**

A) Operational taxonomic units (OTUs) that correlated with PC1 (|cor| > 0.6) highlighted Lachnospiraceae and Ruminococcaceae were associated with later timepoints while the earlier samples contained a greater abundance of Proteobacteria. B) Alpha diversity was calculated at each timepoint using three different metrics (Observed OTUs, Chao1 and Shannon). Groups were compared with the Kruskal-Wallis test. Significance was obtained at enrolment for both observed OTUs and Chao1 metrics and at 3 months only for observed OTUs. Following pairwise comparisons with Wilcoxon test and BH correction, the Reference group displayed a significantly higher alpha diversity compared to Control (Observed OTUs metric, *p* = 0.0056 and Chao1 metric, *p* = 0.0024) at enrolment. Differences between Test and Control groups were close to significance. At 3 months, Reference infants had a significantly lower alpha diversity compared to the Test group (*p* = 0.0016). The Control group had close to significant lower alpha diversity compared to the Test group (*p* = 0.072). No significant differences were observed using the Shannon diversity index. Boxplots represent lower, middle (median) and upper quartile. Significance: · *p* < 0.1, * *p* < 0.05, ** *p* < 0.01. C) Fecal microbial profile differences at the different sampling points. Groups were significantly different at each of the analyzed timepoints (PERMANOVA, *p* < 0.05), with maximum variance observed at 3 months of life, driven by the Reference group. Asterisks represent group centroids.

**Supplementary Table 1.** **Population characteristics of the analyzed samples from MERCURIUS Study.** Study population characteristics for the infants with anthropometry, microbial and metabolic data. Values are reported as percentage or represent the mean of the group ± the standard deviation. Continuous variables were tested with ANOVA, while categorical data were tested pairwise with the Fisher’s exact test (ns = p > 0.05).

|  | **Test**  **(n = 65)** | **Control**  **(n = 61)** | **Reference**  **(n = 38)** | **p value** |
| --- | --- | --- | --- | --- |
| **Delivery Mode** | 68% Vaginal | 77% Vaginal | 76% Vaginal | ns |
| **Gender** | 47% Male | 49% Male | 52% Male | ns |
| **Country of Birth** |  |  |  |  |
| Belgium | 37% | 57% | 39.5% |  |
| Netherlands | 38.5% | 33% | 34% |  |
| France | 9% | 6.5% | 0% |  |
| Singapore | 4.5% | 3% | 26% |  |
| **Ethnicity** |  |  |  |  |
| Caucasian | 81.5% | 93% | 68.5% |  |
| Asian | 6% | 1.5% | 23.5% |  |
| Other | 7.5% | 3.2% | 8% |  |
| **Gestational Age (weeks)** | 39.44 ± 1.10 | 39.29 ± 1.16 | 39.49 ± 1.25 | ns |
| **Weight (g)** | 3359.07 ± 353 | 3295.75 ± 358 | 3342.31 ± 319 | ns |
| **Length (cm)** | 49.70 ± 1.99 | 49.52 ± 1.95 | 49.72 ± 1.90 | ns |
| **Head Circumference (cm)** | 34.41 ± 1.34 | 34.51 ± 1.05 | 34.03 ± 1.25 | ns |
| **Infant Antibiotic Usage** | 74% No | 85% No | 79% No | ns |

**Supplementary Table 2.** VIPs of extracted OTUs influencing PLS-DA model (CER = 0.48) built on Test and Control groups at enrolment.

**Supplementary Table 3.** VIPs of extracted OTUs influencing PLS-DA model (CER = 0.41) built on Test and Control groups at 3 months.

**Supplementary Table 4.** ANCOM-BC2 differential abundance analysis.

**
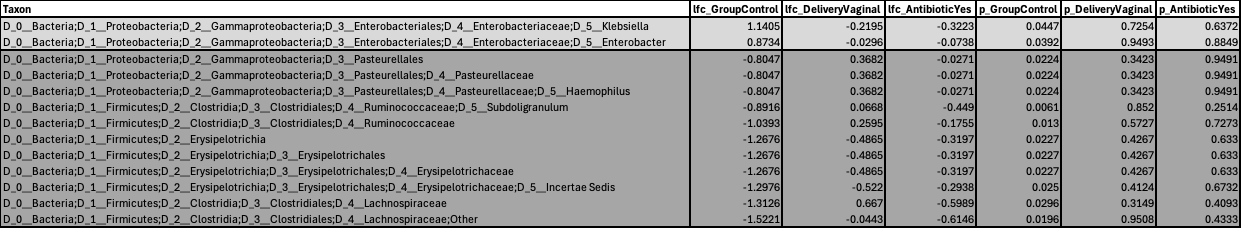
**

**Supplementary Table 6.** Extracted VIP score from pairwise PLS-DA model (CER = 0.08) comparing the Control and Test fecal metabolic profiles measured by UPLC-MS at 3 months of life.

**Supplementary Figure 3:** Metabolic and lipid content of the Test and Control infant formula measured by the liquid chromatography-mass spectrometry.

**Supplementary Table 7.** Fecal metabolism indicators found to significantly differ between Test and Control infants. Wilcoxon rank-sum test was used to calculate the p-values, which were FDR corrected (q = 0.05). A fold change threshold of >2 and <-2 was applied.

**Supplementary Table 8.** Fecal metabolites measured by UPLC-MS commonly different in Reference and Test infants compared to Control infants. Green shaded metabolites indicate metabolites higher in Reference and Test stools compared to Control stools and Orange shaded metabolites highlight those lower.

**Supplementary Table 9.** Extracted VIP score from pairwise PLS-DA model (CER = 0.04) comparing the Test and Reference fecal metabolic profiles at 3 months of life measured by UPLC-MS.

**Supplementary Table 10.** Fecal metabolism indicators found to significantly differ between Test and Reference infants. Wilcoxon rank-sum test was used to calculate the p-values, which were FDR corrected (q = 0.05). A fold change threshold of >2 and <-2 was applied.

**Supplementary Table 11.** Extracted VIP score from pairwise PLS-DA model (CER = 0.05) comparing the Reference and Control fecal metabolic profiles at 3 months of life measured by UPLC-MS.

**Supplementary Table 12.** Fecal metabolism indicators found to significantly differ between Control and Reference infants. Wilcoxon rank-sum test was used to calculate the p-values, which were FDR corrected (q = 0.05). A fold change threshold of >2 and <-2 was applied.

**Supplementary Table 13.** Plasma metabolites measured by UPLC-MS observed to differ in the PLS-DA model between Test and Control infants at 3 months.

**Supplementary Table 14.** Common metabolic variation in the plasma metabolites of Test and Reference infants compared to Control infants at 3 months. Metabolic differences determined by PLS-DA models (VIP > 1).

**Supplementary Table 15.** Plasma metabolites measured by UPLC-MS observed to differ in the PLS-DA model (CER = 0.06) between Test and Reference infants at 3 months.

**Supplementary Table 16.** Plasma metabolites measured by UPLC-MS observed to differ in the PLS-DA model (CER = 0.07) between Control and Reference infants at 3 months.

**Supplementary Table 17.** Plasma metabolism indicators found to significantly differ between Control and Reference infants. Wilcoxon rank-sum test was used to calculate the p-values, which were FDR corrected (q = 0.05). A fold change threshold of >1.5 and <-1.5 was applied.

**Supplementary Table 18.** Plasma metabolism indicators found to significantly differ between Test and Reference infants. Wilcoxon rank-sum test was used to calculate the p-values, which were FDR corrected (q = 0.05). A fold change threshold of >1.5 and <-1.5 was applied.

**Supplementary Table 19.** Plasma metabolism indicators found to significantly differ between Control and Reference infants. Wilcoxon rank-sum test was used to calculate the p-values, which were FDR corrected (q = 0.05). A fold change threshold of >1.5 and <-1.5 was applied.

**Supplementary Table 20.** Plasma and fecal metabolites measured at 3 months significantly correlated with total sum skin thickness measures at one year in all infants. Pearson correlations shown for all features significant after a Benjamini-Hochberg correction (p < 0.1).
